# Acetic acid positively modulates proline metabolism for mitigating PEG-mediated drought stress in Maize and Arabidopsis

**DOI:** 10.3389/fpls.2023.1167238

**Published:** 2023-07-19

**Authors:** Sakil Mahmud, Mohammad Kamruzzaman, Sabarna Bhattacharyya, Khadiga Alharbi, Diaa Abd El Moneim, Mohammad Golam Mostofa

**Affiliations:** ^1^ Department of Biochemistry and Molecular Biology, Bangladesh Agricultural University, Mymensingh, Bangladesh; ^2^ Department of Plant Breeding, Institute of Crop Science and Resource Conservation, University of Bonn, Bonn, Germany; ^3^ Plant Breeding Division, Bangladesh Institute of Nuclear Agriculture (BINA), Mymensingh, Bangladesh; ^4^ Plant Cell Biology, Institute of Cellular and Molecular Botany, University of Bonn, Bonn, Germany; ^5^ Department of Biology, College of Science, Princess Nourah bint Abdulrahman University, Riyadh, Saudi Arabia; ^6^ Department of Plant Production (Genetic Branch), Faculty of Environmental Agricultural Sciences, Arish University, El-Arish, Egypt; ^7^ Department of Energy Plant Research Laboratory, Michigan State University, East Lansing, MI, United States; ^8^ Department of Biochemistry and Molecular Biology, Michigan State University, East Lansing, MI, United States

**Keywords:** acetic acid, antioxidant defense, maize (*Zea mays* L.), osmotic stress, proline metabolism

## Abstract

**Introduction:**

Osmotic imbalance is one of the major consequences of drought stress, negatively affecting plant growth and productivity. Acetic acid has modulatory roles in osmotic balance in plants; however, the mechanistic insights into acetic acid-mediated osmotic adjustment under drought stress remains largely unknown.

**Methods:**

Here, we investigated how seed priming and seedling root treatment with acetic acid enabled maize plants overcoming polyethylene glycol (PEG)-induced drought effects.

**Results:**

Maize seeds primed with acetic acid showed better growth performance when compared with unprimed seeds under PEG application. This growth performance was mainly attributed to improved growth traits, such as fresh weight, dry weight, length of shoots and roots, and several leaf spectral indices, including normalized difference vegetation index (NDVI) and chlorophyll absorption in reflectance index (MCARI). The levels of oxidative stress indicators hydrogen peroxide (H_2_O_2_) and malondialdehyde (MDA) did not alter significantly among the treatments, but proline content as well as the expression of proline biosynthetic gene, *Δ1-PYRROLINE-5-CARBOXYLATE SYNTHETASE 1* (*P5CS1*) was significantly elevated in plants receiving acetic acid under PEG-treatments. On the other hand, treating the seedlings root with acetic acid led to a significant recovery of maize plants from drought-induced wilting. Although growth traits remained unchanged among the treatments, the enhancement of leaf water content, photosynthetic rate, proline level, expression of *P5CS1*, and antioxidant enzyme activities along with reduced level of H_2_O_2_ and MDA in acetic acid-supplemented drought plants indicated a positive regulatory role of acetic acid in maize tolerance to drought. Moreover, the high expression of *P5CS1* and the subsequent elevation of proline level upon acetic acid application were further validated using wild type and proline biosynthetic mutant *p5cs1* of Arabidopsis. Results showed that acetic acid application enabled wild type plants to maintain better phenotypic appearance and recovery from drought stress than *p5cs1* plants, suggesting a crosstalk between acetic acid and proline metabolism in plants under drought stress.

**Discussion:**

Our results highlight the molecular and intrinsic mechanisms of acetic acid conferring plant tolerance to drought stress.

## Introduction

Ever changing environments are often stressful for growth and development of plants. Drought is considered as a major abiotic stress, affecting plant ecological distribution, growth, and productivity worldwide (Gupta et al., 2020). Drought causes osmotic stresses that lead to rapid changes in gene expression and metabolic alterations, some of which help plants to cope with such stresses (Kamruzzaman et al., 2022; Mahmud et al., 2022). Osmotic stress can directly interrupt stomatal regulation, resulting in poor photosynthetic performance in plants (Mittler et al., 2022). Reactive oxygen species (ROS) accumulation is a secondary response in plants under osmotic-related stresses, including water-shortage. However, an imbalance between production and scavenging of ROS, such as hydrogen peroxide (H_2_O_2_), superoxide radicals (O_2_^·−^), singlet oxygen (^1^O_2_), and hydroxyl radicals (HO^•^) may initiate a cascade of oxidative reactions that result in membrane lipid peroxidation, chlorophyll degradation, protein denaturation, and DNA strand breakage (Claeys and Inze, 2013; Lei et al., 2021; Mittler et al., 2022). Plants have evolved sophisticated antioxidant mechanisms to counter the damaging effects of ROS. The antioxidant system includes non-enzymatic compounds, such as tocopherols, glutathione, ascorbate, and carotenoids, as well as enzymes, including catalase (CAT, EC: 1.11.1.6), ascorbate peroxidase (APX, EC: 1.11.1.11), peroxidase (POD, EC: 1.11.1.7), and superoxide dismutase (SOD, EC 1.15.1.1) (Nikoleta-Kleio et al., 2020; Mittler et al., 2022).

Plants accumulate an array of osmolytes to adjust their intra-cellular osmotic potential under stressful conditions. Osmolytes are known to stabilize the protein structure and are ubiquitous in living organisms. The major organic osmolytes include amino acids (e.g., proline), methylamines (e.g., betaine and trimethylamine-N-oxide), polyamines (e.g., spermidine), sugars (e.g., trehalose), and polyols (e.g., sorbitol) (Ozturk et al., 2021; Singh et al., 2021). Among these osmolytes, proline has cardinal importance in plants, both as structural unit of protein and osmolyte for osmoprotection (Moukhtari et al., 2020; Alvarez et al., 2022; Spormann et al., 2023). When plants are exposed to osmotic stress, proline accumulates in the cytoplasm and acts as an osmoprotectant for stabilizing cellular membranes and maintaining turgor pressure. This helps plant prevent damage to cellular components, allowing plants to continue to grow and function maximally despite the stress. In addition to its role as an osmoprotectant, proline has been shown to enhance the expression of stress-responsive genes, which are involved in plant responses to stress (Shrestha et al., 2021; Kamruzzaman et al., 2022). Besides its role as an osmolyte, proline has been further reported to scavenge ROS under drought stress (Moukhtari et al., 2020). Nonetheless, proline accumulation alone cannot provide tolerance rather other pathways activated upon different abiotic stresses are linked to its effect on plants (Chun et al., 2018; Ozturk et al., 2021). Under drought stress, synthesis of proline in the cytosol derived from the precursor glutamate in two-sequential steps catalyzed by δ-pyrroline-5-carboxylate synthetase (P5CS) and δ-pyrroline-5-carboxylate reductase (P5CR) (Adamipour et al., 2020). *P5CS* expression increases upon exposure to drought while low or no increase in *P5CR* expression depending on plant species (Chen et al., 2021).

Maize (*Zea mays* L.) is a major agricultural crop cultivated across the world. Despite having wide genetic variation, most of the maize varieties are moderately sensitive to drought stress, especially seedling stage of maize is more sensitive to water-shortage (Badr et al., 2020; Sheoran et al., 2022). The negative effects of drought stress on maize plants are involved with changes in morphological and physiological parameters, including shoot and root growth, photosynthetic activity, stomatal features, proline accumulation, and abscisic acid levels in the whole plant (Salika and Rifat, 2021; Sheoran et al., 2022). Reports suggest that invisible wilting symptoms induced by high osmotic potential may lead to 60% yield losses in maize (Avramova et al., 2015; Farooq et al., 2015). Thus, developing maize varieties with better osmotic stress tolerance potential continues to be an important strategy in sustaining maize production in arid and semi-arid areas globally. Endogenous level of proline accumulation is enhanced in maize root upon low water potential, which has been reported to be a tolerance mechanism against the osmotic stress (Kang et al., 2022). Besides, exogenously applied proline in maize plants can enhance the antioxidant activity, better growth performance, and prevent water loss upon osmotic stresses induced by drought and salinity (Mosaad et al., 2020; Ibrahim et al., 2022). Polyethylene glycol (PEG)-mediated elevation of osmotic potential inhibits apical shoot growth while increasing lateral root formation, suggesting a potential mechanism of maize plants to combat water deficiency (Ji et al., 2014).

Recently, there has been increasing interest in using plant growth-promoting compounds, such as acetic acid, to mitigate osmotic stress in plants. Acetic acid is a naturally occurring organic acid that has been shown to have various positive effects on plant growth and stress tolerance by improving water and nutrient uptake and photosynthesis rate (Kim et al., 2017; Rahman et al., 2019; Mendiburu, 2019). Several studies reported the potential aspects of acetic acid in enhancing plant resistance to a variety of abiotic stresses, including salinity and drought. For example, application of acetic acid potentiated drought tolerance mechanisms in cereal crops rice (*Oryza sativa*), maize, and wheat (*Triticum aestivum*) through the activation of various physiological and molecular mechanisms (Kim et al., 2017; Ogawa et al., 2021). A low dose (<50 mM) of acetic acid has been suggested to be effective in mitigating drought adversities in cassava (*Manihot esculenta*) (Utsumi et al., 2019) and mung bean (*Vigna radiata*) (Rahman et al., 2019). Although the beneficial effects of acetic acid on osmotic stress tolerance are already quite recognized, the regulatory mechanism is still not fully understood. Moreover, acetic acid-induced proline accumulation varies upon different plants and abiotic stresses. For example, proline level was elevated with the application of acetic acid under salt stress in mung bean (Rahman et al., 2019), whereas the level was lowered upon drought stress in soybean (*Glycine max*) (Rahman et al., 2020). Thus, how acetic acid application regulates cellular level of proline for rendering osmotic stress tolerance is yet to be studied at genetic level in plants.

In the current study, we attempted to explore the functions of acetic acid in mitigation of PEG-induced osmotic stress by studying several morphological, physiological, and biochemical features in maize. We compared the seed priming and seedling treatment modes of acetic acid to understand common pathway(s) elicited by acetic acid for conferring drought tolerance in maize. We also validated our findings by conducting a complementary functional study using the model plant *A. thaliana* wild-type and its proline biosynthetic mutant *p5cs1*. Our comprehensive study using both maize and Arabidopsis provided an in-depth understanding of how acetic acid modulates cellular intrinsic mechanisms to make plants more resilient to osmotic stress.

## Materials and methods

### Experimental design and treatments

The seeds of maize (*Zea mays* L., cv Baden seed corn; kindly provided by Kiepenkerl, Blooming, Germany) were surface sterilized with 70% (v/v) ethanol treatment for 1 min followed by 0.5% (v/v) sodium hypochlorite solution for 10 minutes. Seeds were then washed 5 times with distilled water (dH_2_O). For priming, the treatments were as follows: Ctrl (Control, dH_2_O), PEG (20% w/v polyethylene glycol-6000), AA (500 μM acetic acid), and PEG + AA (20% w/v PEG and 500 μM AA). The dose of AA (500 μM) was selected based on the trial with the different concentrations of AA that did not negatively impact the germination percentage without influencing proline content (**Supplementary Figures 1A–C**). Seeds were soaked overnight with above-mentioned solutions and allowed for germination on a wet filter paper. Growth assessment and biochemical analyses of germinated seedlings were performed after growing in commercial hydroponic nutrient solution (Flora Series® nutrient system: Floragro® (total nitrogen (N): 2%, available phosphate (P_2_O_5_): 1%, soluble potash (K_2_O): 6%, magnesium (Mg): 0.5%), Floramicro® (total nitrogen (N): 5%, soluble potash (K_2_O): 1%, calcium (Ca): 5%, boron (B): 0.01%, cobalt (Co): 0.0005%, copper (Cu): 0.01%, iron (Fe): 0.1%, manganese (Mn): 0.05%, molybdenum (Mo): 0.0008%, zinc (Zn): 0.015%) and Florabloom® (available phosphate (P_2_O_5_): 5%, soluble potash (K_2_O): 4%, magnesium (Mg): 1.5%, sulfur (S): 1%), USA, 2 ml/2 L distilled water) for 10 days. All the above experiments were repeated three times to ensure the reproducibility of the experiment.Similarly, the treatments for root application at the seedlings stage were Ctrl (hydroponic nutrient solution), PEG (20% w/v), AA (25 mM), and PEG + AA (treatment with 20% w/v PEG and 25 mM AA). The dose of acetic acid (25 mM) was selected based on the findings of Kim et al. (2017). Solution of ‘Ctrl’ treatment was used as a solvent to prepare other treatments. Properly germinated seedlings in the wet filter paper were grown for 12 days in ‘Ctrl’ solution followed by subjecting to different treatments for 48 h. After the treatment period, leaves were harvested to assess various morphological, physiological, and biochemical parameters. Morphological parameters included shoot fresh weight (SFW), root fresh weight (RFW), shoot dry weight (SDW), root dry weight (RDW), root length (RL), shoot length (SL), root volume, and average root diameter. The biochemical parameters were proline, MDA, H_2_O_2_, CAT, POD, and APX.

**Figure 1:**
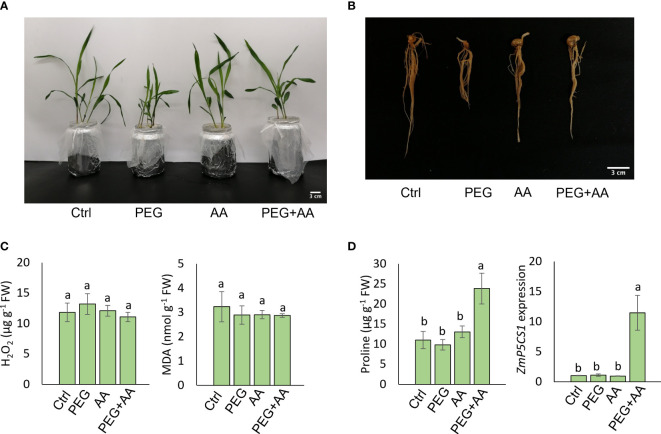
Seed priming effect of acetic acid on maize growth and oxidative stress parameters in response to PEG treatments. **(A, B)** Phenotype of shoots **(A)** and roots **(B)** of maize plants grown for 10 days after seed priming with the treatments for overnight. **(C, D)** The levels of oxidative stress markers hydrogen peroxide (H_2_O_2_) and malondialdehyde (MDA) **(C)**, and proline content and relative expression of *ZmP5CS1* **(D)** in 10 days old maize leaves recorded after seed priming. Data represent mean ± SE of six individual replicates (n=6) for H_2_O_2_, MDA and proline content and three individual replicates (n=3) for *ZmP5CS1* expression, and different alphabetical letters indicate significant variations among the treatments following Tukey’s *Post-Hoc* HSD test (*P*<0.05). FW, fresh weight. Ctrl, PEG (20%), AA (500 μM), and PEG+AA represent treatments with distilled water (dH_2_O), 20% polyethylene glycol, 500 μM acetic acid, and 20% polyethylene glycol + 500 μM acetic acid, respectively. FW, fresh weight.

In both priming and exogenous treatment experiments, plants were grown in a growth chamber (Bronson Climate, LW Zaltbommel, the Netherlands) set at 26° C with 12/12 h dark/light cycle in white fluorescent light up to 600 μmol m^−2^ s^−1^ and 55-60% relative humidity. A handmade system, made of nylon mesh and glass jar, was adopted for seedlings growth in this study (**Figures 1A, C**). The treatment solutions were replaced in every 48 h on a regular basis. All of the above experiments were repeated three times.

For Arabidopsis (*A. thaliana*) experiment, wild-type Columbia ecotype (Col-0) and *p5cs1* T-DNA insertion mutant seeds (Funck et al., 2012) were surface sterilized using 5.25% sodium hypochlorite for 1 min and then washed 5 times with sterilized dH_2_O. Sterilized seeds were placed on the petri-plates containing Murashige and Skoog medium (MS; Duchefa Biochemie) with 1% (w/v) sucrose and 0.6% (w/v) phytagel (Sigma‐Aldrich, Inc.) for germination and properly germinated seedlings were grown for 14 days. Properly grown plants were then placed in liquid culture (Rivero et al., 2013) containing different treatments in a sterile plastic box with gentle rotation (120 rpm) on an orbital shaker (Innova 2000, New Brunswick Scientific, USA). The dose of AA (500 μM) was selected based upon trial with the different concentrations of acetic acid and that did not negatively impact the germination percentage with a steady increase of proline content compared to non-treated plants (**Supplementary Figures 2A, B**). Immediately, after 48 h of treatment, seedlings were re-placed into petri-plates containing MS with 1% (w/v) sucrose and 0.6% (w/v) phytagel and were grown for another seven days to observe the growth. For biochemical analysis, the whole shoot of the plants was collected immediately after 48 h of treatment in the liquid culture. If not otherwise stated, plants were cultured in growth chambers with long day conditions (16 h/8 h light/dark) at 22°C and up to 100 µmol photons m^-2^ s^-1^ and 60% relative humidity. Phenotypic experiment was repeated two times and biochemical assays were repeated three times.

### Assessment of morphological traits and leaf spectral indices

Root and shoot fresh weights were recorded by a digital balance and then dried into an oven at 70°C for 3 days to measure the dry weights. Leaf water status was estimated through relative water content (RWC) according to Ghoulam et al. (2002). Tip of flag leaves were cut into 5-6 small pieces and the fresh weight (FW) was recorded. Then, the leaf pieces were dipped into 10 mL deionized water into a falcon tube for 24 h at room temperature. After 24 h, the leaves were wiped with a paper towel and the turgid weight (TW) was recorded. Dry weight was recorded after oven drying at 70°C for 72 h. RWC was estimated as (FW-DW)/(TW-DW) ×100. Three measurements were taken from each leaf. A portable infrared gas exchange analyzer (LI-6400 XT; LI-COR) was used (at 15:00 pm, when light was on) to measure the photosynthetic parameters, namely stomatal conductance (*g_s_*), transpiration rate (*E*), intercellular CO_2_ concentration (*Ci*), and photosynthesis rate (*A*) from flag leaves according to previous studies (Siddiqui et al., 2021; Fang et al., 2022). The internal ambient CO_2_ level was 400 ppm.

Leaf spectral reflectance was also measured on the flag leaves with at least three plants using a PolyPen RP410 device (PSI, Drasov). Three points were measured from each leaf of each plant, and then the average of the three points was computed as described in previous study by Begum et al. (2020). Among various vegetation indices, Normalized Difference Vegetation Index (NDVI) = (RNIR − RRED)/(RNIR + RRED) (Rouse et al., 1973);, Chlorophyll Absorption in Reflectance Index (MCARI) = [(R700 − R670) − 0.2 * (R700 − R550)] x (R700/R670) (Daughtry et al., 2000), Simple Ratio Pigment Index (SPRI), Carter Indices (Ctr1), Lichtenthaler Indices (Lic2), Greenness Index (GI), Photochemical Reflectance Index (PRI) and Normalized Pigment Chlorophyll Index (NPCI) were recorded. In all equations, R indicates the reflectance at a given wavelength of light. Roots were scanned using an Epson scanner (Perfection LA24000) with a resolution of 600 dots per inch and root images were analyzed using the WinRhizo software (Regent Instruments Inc., Quebec, Canada) to record the root architectural traits including average root diameter (mm), and root volume (cm^3^) from 6 individuals. SFW, SDW, RFW, RDW, SL, and RL were averaged from 6 individual replicates. A normal measuring ruler was used to measure the lengths.

### Proline, MDA, and H_2_O_2_ determination

First, fully expanded leaf of six maize and whole shoot of six Arabidopsis were used for proline, MDA, and H_2_O_2_ determination. Proline was determined according to the method described by Bates et al. (1973). Samples were ground in liquid nitrogen and 90-100 mg powdered tissue was homogenized in 1.5 mL of 3% sulphosalicylic acid (w/v). The suspension was centrifuged at 12,000 g for 5 min and 200 µL of sample supernatant was added with 200 µL acetic acid and 200 µL ninhydrin reagents. This mixture was incubated at 95°C temperature for 60 min and the reaction was immediately stopped by putting it into ice for 3-5 min. Then, 600 µL of pure toluene was mixed and incubated at room temperature for 30 min. The chromatophore readings were recorded at 520 nm wavelength with 10 reads per well through 96 well plastic plates using a microplate reader (TECAN Infinite 200 Pro, TECAN Group Limited, Switzerland). Proline content was estimated using standard curve method and expressed in µg/g FW.

Hydrogen peroxide (H_2_O_2_) was determined according to the previously described method (Sergiev et al., 1997; Velikova et al., 2000). Samples were grounded into powder using liquid nitrogen and 90-100 mg powder was homogenized into 500 µL 0.1% (w/v) trichloroacetic acid (TCA) and centrifuged at 12,000 g for 10 min. Then, 200 µL supernatant was added to 200 µL of 10 mM potassium phosphate buffer (pH 7.0 and 1 M 400 µL potassium iodide and mixed through vortexing. The sample absorbance was recorded at 390 nm through a microplate reader (TECAN Infinite 200 Pro, TECAN Group Limited, Switzerland). H_2_O_2_ level was determined by developing standard curve with known concentration of H_2_O_2_ and expressed in µg/g FW.

Lipid membrane damage was estimated by determining malondialdehyde (MDA) content using thiobarbituric acid (TBA) method (Hodges et al., 1999) adapted to a microplate-based protocol. Samples were homogenized in liquid nitrogen and 100 mg of pulverized powder was added to 1.5 mL of 0.1% (w/v) trichloroacetic acid (TCA) followed by centrifugation at 14,000 g for 15 min at 4°C. Then, 500 μl supernatant was mixed with reaction solution I (0.01% w/v 2,6-di-tert-butyl-4-methyl phenol (BHT) in 20% w/v TCA) and reaction solution II (0.65% w/v TBA, 0.01% w/v BHT in 20% w/v TCA) in a 1:1 ratio. The sample mix was then incubated at 95°C for 30 min. The reaction was stopped by putting on ice for five minutes, and the reaction mix was centrifuged at 8000 g for 10 min at 4°C. The absorbance of the supernatant was measured at 440, 532, and 600 nm using a microplate reader (TECAN Infinite 200 Pro, TECAN Group Limited, Switzerland). MDA content was expressed as nanomoles per gFW.

### Assay of antioxidant enzyme activity

Leaf samples (500 mg/sample) were ground into powder through liquid nitrogen and then homogenized into 1 mL of 50 mM ice-cold potassium phosphate (K-P) buffer, pH 7.0 (100 mM potassium chloride (w/v), 5 mM β-mercaptoethanol and 10% glycerol v/v). The homogenized tissue was centrifugated at 11,500 g for 2 min, and the collected supernatant was used for measuring protein content and enzyme activities.

To determine the activity of catalase (CAT, EC: 1.11.1.6), we monitored the decline of absorbance for 1 minute at 240 nm, following the method of Rahman et al. (2019). The reaction mixture consisted of K-P buffer (50 mM, pH 7.0), H_2_O_2_ (15 mM), and enzyme extract (5 μL), with a final volume of 700 μL. CAT activity was calculated using an extinction coefficient of 39.4 M^-1^ cm^-1^.

The activity of APX was assessed according to the method of Nakano and Asada, 1981 by recording absorbance change at 290 nm for 1 minute. The reaction mixture contained K-P buffer (50 mM, pH 7.0), ascorbate (AsA, w/v) (0.5 mM), ethylenediaminetetraacetic acid (EDTA, 0.1 mM), H_2_O_2_ (0.1 mM), and enzyme extract (5 μL), with a final volume of 700 μL. APX activity was estimated using an extinction coefficient of 2.8 M^-1^ cm^-1^.

The activity of peroxidase was assessed using the method of Hemeda and Klein (1990). The reaction mixture contained K-P buffer (25 mM, pH 7.0), guaiacol (0.05%, v/v), H_2_O_2_ (10 mM), and enzyme extract (5 μL), with a final volume of 700 μL. An increase in absorbance was recorded at 470 nm for 1 min, and the extinction coefficient of 26.6 mM^-1^ cm^-1^ was used to calculate POD activity.

Total protein from the enzyme extract was measured using Coomassie Bradford protein assay kit (cat. 23200, Thermo Scientific USA) according to manufacturer’s instructions.

### *P5CS1* expression analysis in maize and Arabidopsis using RT-qPCR

Fully expanded leaves of three maize and whole shoot of three Arabidopsis seedlings were homogenized in liquid nitrogen, and RNA was extracted using Monarch RNA miniprep kit (New England Biolabs, USA) following the manufacturer’s instruction. The RNA concentration and quality were determined by a nanodrop (NanoDrop 2000c, Thermo Fischer Scientific, USA). cDNA was synthesized using Luna Script super RT mix (New England Biolabs, USA) following the manufacturer’s instruction. Quantitative real-time PCR (RT-qPCR) was performed in 96-well plates using a 7500 fast real-time PCR system (Applied Biosystems, USA). A SYBR green-based Luna Universal qPCR master mix was used in the assay with three technical replicates per sample. The qPCR run was set to initial denaturation at 95°C for 3 min followed by 40 cycles (95°C for 15 s, 60°C for 1 min). Specific amplification was analyzed using a melt curve (95°C for 15 s, 60°C for 1 min, 95°C for 15 s). Primers were designed by using the Oligo Calculator, version 3.27 (http://biotools.nubic.northwestern.edu/OligoCalc.html). Primer efficiency was estimated before running the qPCR reaction (0.936 for *Actin2* and 0.946 for *AtP5CS1* in Arabidopsis, and 0.921 for 19 *α-zein* and 0.932 for *ZmP5CS1* in maize). Relative mRNA expression of *P5CS1* was normalized to the reference, 19 *α-zein* in maize and *Actin2* in Arabidopsis and calculated based on the 2^− ΔΔCt^ method. Primers used in the study are listed in the **Supplementary Table 2**.

### Statistical analysis

Statistical significance was analyzed using open-access statistical computing and statistical platform RStudio (version 4.0.3). All the statistical analysis was performed with one-way ANOVA followed byTukey’s PostHoc HSD test except a two-way ANOVA for **Figure 1D**. For one-way ANOVA and Tukey’s PostHoc HSD test the package agricolae (Mendiburu, 2019) and tidyverse (Wickham, 2021), respectively, were used. All codes were designed on a R script (version 4.0.3). Bar plots with error bars were generated in Microsoft Excel (version 16.69.1).

## Results

### Maize seed priming

#### Seed priming with acetic acid improved morphological attributes of PEG-primed maize plants

Maize seeds were primed with 500 μM acetic acid (AA) or/and with PEG (PEG+AA) and germinated seedlings were grown in the non-treated hydroponic solutions to observe the priming effect of acetic acid under drought stress. Our observations revealed stunted shoot and root growth in plants treated with PEG, while no visible differences were observed in ‘AA’ compared to ‘Ctrl’ (**Figures 1A, B**). However, a similar pattern of shoot and root growth was observed between ‘PEG+AA’ and ‘Ctrl’ (**Figures 1A, B**). The shoot and root growth parameters, specifically SFW, SDW, RFW, and RDW were significantly decreased by 22%, 20%, 40%, and 43%, respectively, under ‘PEG’ compared with ‘Ctrl’. However, these reductions were recovered in ‘PEG+AA’, where SFW, SDW, RFW, and RDW showed a decrease of 12%, 5% (not significant), 19%, and 13%, respectively, compared with ‘Ctrl’ (**Table 1**). The growth parameters in ‘AA’ did not exhibit significant variation when compared to the ‘Ctrl’, which is consistent with the results of visual observations.

**Table 1:** Effect of acetic acid on leaf spectral reflectance indices of maize plants subjected to seed primed PEG treatments*.

Parameters		Ctrl	PEG	AA	PEG+AA
**SFW (mg)**	Mean	1022.17^a^	792.17^b^	1043.17^a^	899.50^ab^
	SD	163.42	27.19	163.55	22.32
**RFW (mg)**	Mean	609.66^a^	364.17^b^	550.33^a^	490.66^ab^
	SD	38.87	22.48	95.68	182.22
**SDW (mg)**	Mean	80.33^a^	64.50^b^	81.33^a^	75.83^a^
	SD	8.43	7.40	4.84	3.87
**RDW (mg)**	Mean	44.43^a^	25.28^b^	41.32^a^	38.51^ab^
	SD	1.02	0.95	2.05	1.75
**SL (cm)**	Mean	34.50^a^	24.60^b^	32.66^a^	34.16^a^
	SD	2.35	3.76	1.75	2.86
**RL (cm)**	Mean	15^a^	8.83^b^	14.17^a^	14.83^a^
	SD	3.03	1.47	2.32	3.49
**Total root volume (cm^3^)**	Mean	0.289^b^	0.45^a^	0.28^b^	0.33^b^
	SD	0.07	0.04	0.05	0.07
**Total root** **avg. diam (mm)**	Mean	0.82^b^	0.98^a^	0.80^b^	0.89^b^
	SD	0.06	0.065	0.02	0.06
**RWC (%)**	Mean	97.59^a^	96.60^a^	96.72^a^	96.53^a^
	SD	0.39	0.978	1.77	0.52
**NDVI**	Mean	0.57^a^	0.38^b^	0.58^a^	0.55^a^
	SD	0.03	0.01	0.01	0.01
**MCARI**	Mean	0.10^b^	0.16^a^	0.10^b^	0.10^b^
	SD	0.001	0.0077	0.0103	0.004
**SPRI**	Mean	0.98^a^	0.75^b^	0.97^a^	0.97^a^
	SD	0.014	0.015	0.022	0.016
**Ctr1**	Mean	1.27^b^	1.56^a^	1.30^b^	1.33^b^
	SD	0.05	0.028	0.082	0.05
**Lic2**	Mean	0.89^b^	0.99^a^	0.86^bc^	0.82^c^
	SD	0.022	0.081	0.017	0.034
**Germination Percentage**	Mean	100^a^	99^a^	99^a^	100^a^
	SD	3	2	4	5

*Data represented as mean, standard deviation (SD) of six individual replicates along with the alphabetical letters representing different levels of statistical significance calculated with one-way ANOVA and Tukey‘s Post-Hoc HSD test (P<0.05). SFW, shoot fresh weight; RFW, root fresh weight; SDW, shoot dry weight; RDW, root dry weight; SL, shoot length; RL, root length; RWC, relative water content; NDVI, Normalized Difference Vegetation Index; MCARI, Chlorophyll Absorption in Reflectance Index; SPRI, Simple Ratio Pigment Index; Ctr1, Carter Indices; Lic2, Lichtenthaler Indices.

Under ‘PEG’, both shoot and root length showed a significant reduction of 29% and 41%, respectively, when compared to ‘Ctrl’. However, no significant changes were observed among ‘PEG+AA’, ‘AA’, and ‘Ctrl’ (**Table 1**). Interestingly, the trend was opposite for total root volume and average diameter, which showed a significant increase of 55% and 20%, respectively, in ‘PEG’ compared to ‘Ctrl’. Nevertheless, there were no noticeable differences observed among ‘PEG+AA’, ‘AA’, and ‘Ctrl’ for these parameters.

#### Seed priming with acetic acid improved leaf spectral indices of PEG-primed maize plants

Plants were also studied for leaf spectral reflectance indices. Out of several parameters detected, significant differences were found in Normalized Difference Vegetation Index (NDVI), Simple Ratio Pigment Index (SPRI), Chlorophyll Absorption in Reflectance Index (MCARI), Carter Indices (Ctr1) and Lichtenthaler Indices (Lic2) in the ‘PEG’ compared to ‘Ctrl’ (**Table 1**). NDVI and SPRI significantly decreased by 33% and 23%, respectively, in the ‘PEG’. In contrast, MCARI, Ctr1 and Lic2 showed significant increase of 60%, 22% and 11%, respectively, in the ‘PEG’. However, there was no significant variations among ‘PEG+AA’, ‘AA’, and ‘Ctrl’ for these indices except slightly decrease of Lic2 in ‘PEG+AA’ and ‘AA’ compared with ‘Ctrl’ (**Table 1**). Under ‘PEG+AA’ and ‘AA’, Lic2 was decreased by 8% and 3%, respectively, as compared with ‘Ctrl’.

#### Seed priming with acetic acid stimulated the proline content of PEG-primed maize plants without influencing the oxidative stress indicators

To monitor the plant status under different conditions, oxidative stress related biochemical and physiological parameters were assessed. Here we observed no significant differences in H_2_O_2_ and MDA levels among the treatments (**Figure 1C**), but interestingly, the proline content was highest in ‘PEG+AA’ and significantly increased by 110% as compared with ‘Ctrl’ while proline accumulation in other treatments remained unchanged (**Figure 1D**). In line that, the expression of the proline biosynthetic gene *ZmP5CS1* did not show significant differences among the treatments ‘Ctrl’, ‘AA’, and ‘PEG’, but it was approximately 10-fold higher in ‘PEG+AA’ compared to the ‘Ctrl’ and other treatments (**Figure 1D**). The RWC of shoot showed no variation among the treatments (**Table 1**).

Overall, our data indicates that pre-treating seeds with acetic acid can modify the response of several morphological and biochemical attributes to drought stress in comparison with non-treated plants.

### Exogenous root treatment of maize seedlings

#### Exogenous acetic acid reduced PEG-mediated negative effects on water content

**Figure 2:**
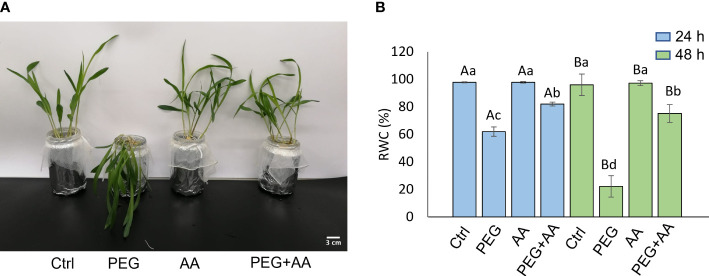
Effects of acetic acid on growth and water content of exogenously treated maize seedlings exposed to PEG-treatments. **(A, B)** Shoot growth **(A)** and relative water content (RWC) **(B)** of maize plants treated with exogenous acetic acid under PEG-treatment. Shoot phenotype was photographed after 24 h of treatments, whereas RWC was recorded after 24 h and 48 h of treatments. Data represent mean ± SE of six individual replicates (n=6) and different alphabetical letters indicate significant variations among the treatments following two-way ANOVA including Tukey’s *Post-Hoc* HSD test (*P*<0.05). Ctrl, PEG (20%), AA (25 mM), and PEG+AA represent treatments with distilled water (dH_2_O), 20% polyethylene glycol, 25 mM acetic acid, and 20% polyethylene glycol + 25 mM acetic acid, respectively.

The above-mentioned morphological study was further devised with exogenous application through root uptake in the 12 days old non-treated seedlings. PEG-treated plants showed severe wilting symptoms in 48 hours after the treatment (**Figure 2A**). As similar to priming plants, exogenous application of acetic acid didn’t employ any visible difference. Intriguingly, the wilting nature of PEG-treatment was recovered in the ‘PEG + AA’ (**Figure 2A**). The severity of PEG-treatment was further demonstrated by measuring the shoot RWC. After 48 h of treating the plants, RWC was declined nearly to 60% in ‘PEG’ while it remained nearly 98% both in ‘Ctrl’ and ‘AA’ (**Figure 2B**). However, the rate of decline was lower (80%) in the ‘PEG+AA’ (**Figure 2B**). Moreover, RWC after 4 days declined to 20% in ‘PEG’. While no variation was found in ‘AA’, the water content was still nearly 60% in the ‘PEG+AA’ (**Figure 2B**).

#### Exogenous acetic acid reduced PEG-mediated negative effects on photosynthesis

**Figure 3:**
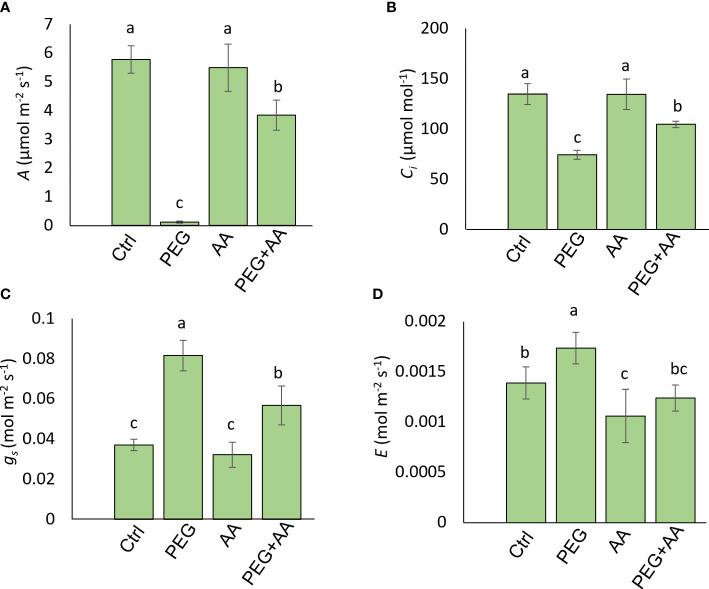
Effects of acetic acid on photosynthetic attributes of exogenously treated maize seedlings exposed to PEG-treatments. **(A-D)**, Photosynthetic parameters, including photosynthetic rate (*A*) **(A)**, internal CO_2_ concentration (*Ci*) **(B)**, stomatal conductance (*g_s_*), **(C)** and transpiration rate (*E*) **(D)** were recorded in maize leaves after 48 h of treatments. Data represent mean ± SE of six individual replicates (n=6) and different alphabetical letters indicate significant variations among the treatments following Tukey’s *Post-Hoc* HSD test (*P*<0.05). Ctrl, PEG (20%), AA (25 mM), and PEG+AA represent treatments with distilled water (dH_2_O), 20% polyethylene glycol, 25 mM acetic acid, and 20% polyethylene glycol + 25 mM acetic acid, respectively.

Two photosynthetic parameters- *A* and *Ci* were significantly reduced by 98% and 45% respectively, in ‘PEG’. However, there were no significant variation found among ‘Ctrl’, ‘AA’ and ‘PEG+AA’ (**Figures 3A, B**). Transpiration related parameters *g_s_* and *E* followed a reciprocal trend compared to photosynthetic parameters with significant up-regulation by 120% and 25% respectively in ‘PEG’ (**Figures 3C, D**). Similar to photosynthetic parameters, no significant variation was found among ‘Ctrl’, ‘AA’ and ‘PEG+AA’. (**Figures 3A, B**). However, the leaf spectral reflectance indices those showed variation in the seed priming plants were remained unaltered albeit changes of three indices- Photochemical Reflectance Index (PRI), Normalized Pigment Chlorophyll Index (NPCI) and Greenness Index (GI) (**Supplementary Table 1**) in the ‘PEG’. Significant decrease of PRI (70%) and GI (17%) was observed in ‘PEG’ compared to ‘Ctrl’. No significant alteration of PRI and GI were found in ‘AA’ and ‘PEG+AA’ compared to ‘Ctrl’ (**Supplementary Table 1**). NPCI showed a significant increase (85%) in the ‘PEG’ compared with ‘Ctrl’. However, the indices had no significant variation in ‘AA’ and ‘PEG+AA’ compared with ‘Ctrl’ (**Supplementary Table 1**).

#### Exogenous acetic acid stimulated antioxidant activity to scavenge ROS

**Figure 4:**
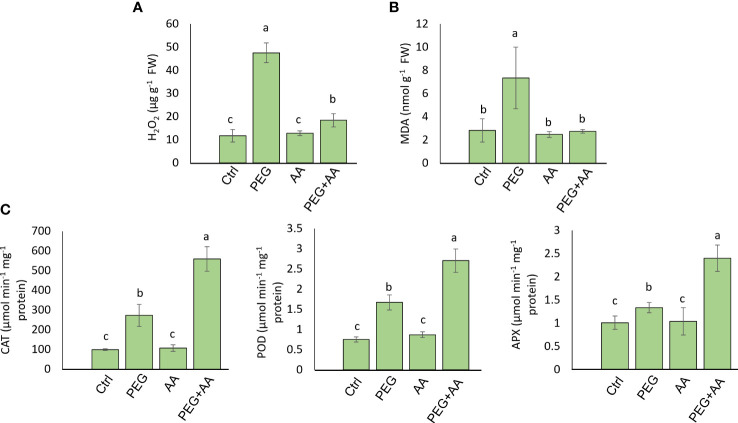
Effects of acetic acid on oxidative stress parameters and antioxidant enzyme activities of exogenously treated maize seedlings exposed to PEG-treatments**. (A, B)** The levels of oxidative stress markers hydrogen peroxide (H_2_O_2_) **(A)** and malondialdehyde (MDA) **(B)**, and the activities of catalase (CAT), ascorbate peroxidase (APX) and peroxidase (POD) **(C)** in the maize leaves were recorded after 48 h of treatments. Data represent mean ± SE of six individual replicates (n=6) and different alphabetical letters indicate significant variations among the treatments following Tukey’s *Post-Hoc* HSD test (*P*<0.05). Ctrl, PEG (20%), AA (25 mM), and PEG+AA represent treatments with distilled water (dH_2_O), 20% polyethylene glycol, 25 mM acetic acid, and 20% polyethylene glycol + 25 mM acetic acid, respectively. FW, fresh weight.

We measured the content of H_2_O_2_, MDA, and proline content in the different treatments. H_2_O_2_ content was abruptly increased in the ‘PEG’ by 304% compared to ‘Ctrl’ (**Figure 4A**). This substantial increase was declined almost nearly to the level of ‘Ctrl’ in the ‘PEG+AA’ and no significant variation found in the ‘AA’ compared to ‘Ctrl’. MDA content increased significantly by 160% upon ‘PEG’ while no significant variation was found in ‘AA’ and ‘PEG+AA’ compared to ‘Ctrl’ (**Figure 4B**). On antioxidant activity furthermore, we measured the catalytic activity of 3 antioxidant enzymes. CAT, POD and APX activity was significantly increased by 176%, 121% and 31% respectively in the ‘PEG’. However, although there was no significant variation in ‘AA’, all of them had significantly higher activity by 466%, 260% and 137% in the ‘PEG+AA’ compared to ‘Ctrl’ (**Figure 4C**).

**Figure 5:**
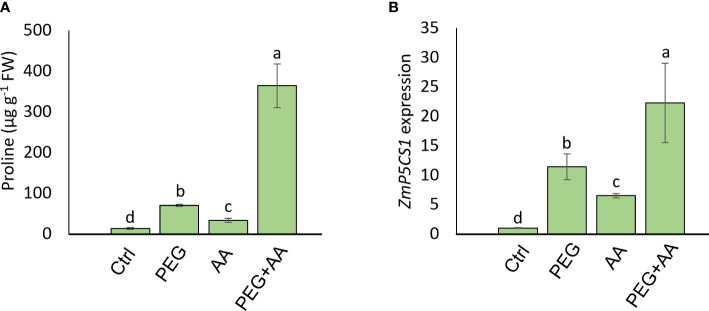
Effects of acetic acid on proline accumulation and transcript level of *ZmP5CS1* of exogenously treated maize seedlings exposed to PEG-treatments**. (A, B)** proline content **(A)** and relative expression of *ZmP5CS1* **(B)** in maize leaves recorded after 48 h of treatments. Data represent mean ± SE of six individual replicates (n=6) and three individual replicates (n=3) for proline and *ZmP5CS1* expression respectively, and different alphabetical letters indicate significant variations among the treatments following Tukey’s *Post-Hoc* HSD test (*P*<0.05). FW, fresh weight. Ctrl, PEG (20%), AA (25 mM), and PEG+AA represent treatments with distilled water (dH_2_O), 20% polyethylene glycol, 25 mM acetic acid, and 20% polyethylene glycol + 25 mM acetic acid, respectively. FW, fresh weight.

Moreover, substantial increase (416%) of proline content was observed in the ‘PEG’ compared to ‘Ctrl’ (**Figure 5A**). Intriguingly, the content was significantly higher by 148% in ‘AA’ and an abrupt increase of 2560% in ‘PEG+AA’ was observed compared ‘Ctrl’. To extend our observations on proline biosynthesis, we checked the expression of *ZmP5CS1*, responsible for the biosynthesis of proline (**Figure 5B**). Expression followed a similar trend to proline content with the increase of around 10-, 4-and 20-fold in the ‘PEG’, ‘AA’ and ‘PEG+AA’, respectively compared to ‘Ctrl’.

Overall, our data indicates that exogenous root treatment with acetic acid can ameliorate the negative effects of drought stress by positively regulating the antioxidant activity.

### Arabidopsis

#### Acetic acid regulated ROS and proline levels in Arabidopsis similar to maize

**Figure 6:**
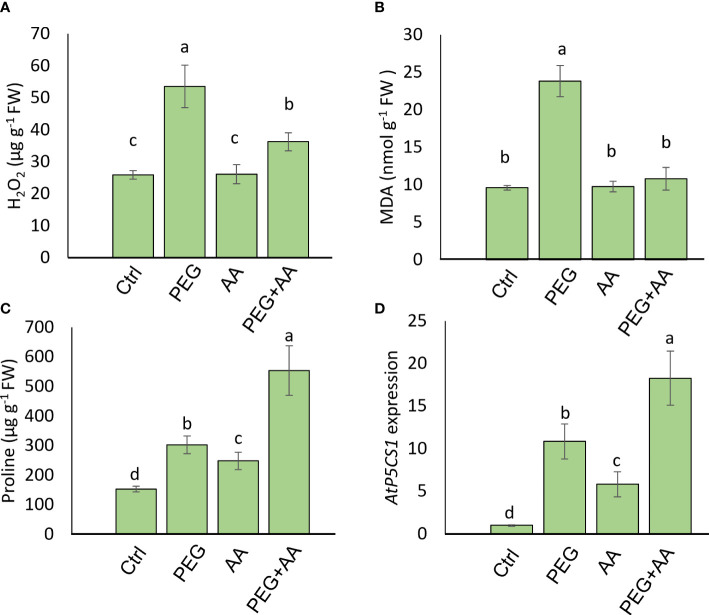
Effects of acetic acid on the accumulation of oxidative stress parameters, proline accumulation, and the transcript level of *AtP5CS1* in Arabidopsis plants exposed to PEG-treatments. **(A, B)** The levels of oxidative stress markers hydrogen peroxide (H_2_O_2_) **(A)** and malondialdehyde (MDA) **(B)**, and **(C, D)** proline content **(C)** and relative expression of *AtP5CS1* **(D)** in Arabidopsis leaves were recorded after 48 h of treatments. Data represent mean ± SE of six individual replicates (n=6) for H2O2, MDA and proline content and three individual replicates (n=3) for *AtP5CS1* expression, and different alphabetical letters indicate significant variations among the treatments following Tukey’s *Post-Hoc* HSD test (*P*<0.05). FW, fresh weight. Ctrl, PEG (20%), AA (500 μM), and PEG+AA represent treatments with distilled water (dH_2_O), 20% polyethylene glycol, 500 μM acetic acid, and 20% polyethylene glycol + 500 μM acetic acid, respectively. FW, fresh weight.

To observe the regulation of proline biosynthesis by acetic acid we further analyzed H_2_O_2_, proline and MDA accumulation in the model plant Arabidopsis under above-mentioned treatments. Similar to the trend of maize plant, H_2_O_2_ content followed an significant increase of 106% in ‘PEG’ compared with ‘Ctrl’. Although there was no significant variation in ‘AA’ but 40% increase was found in ‘PEG+AA’ compared to ‘Ctrl’ that is almost 2-fold lower than ‘PEG’ (**Figure 6A**). MDA content was also enhanced significantly by 148% upon ‘PEG’ while no variation found among ‘AA’, ‘PEG+AA’ and ‘Ctrl’ (**Figure 6B**). Moreover, proline content was significantly increased by 98% in the ‘PEG’ while the increase was even higher (98%) in the ‘PEG+AA’ compared to ‘Ctrl’ (**Figure 6C**). A slight but significant increase of 62% in ‘AA’ was found compared with ‘Ctrl’ which is similar to the observation in maize. Moreover, the observation was confirmed through similar 10-, 5- and 20-fold higher expression of proline biosynthetic gene *AtP5CS1* in ‘PEG’, ‘AA’ and ‘PEG+AA’ respectively compared with ‘Ctrl’ (**Figure 6D**).

#### Functional validation of Arabidopsis *P5CS1* in acetic acid-mediated proline accumulation under PEG-treatment

**Figure 7:**
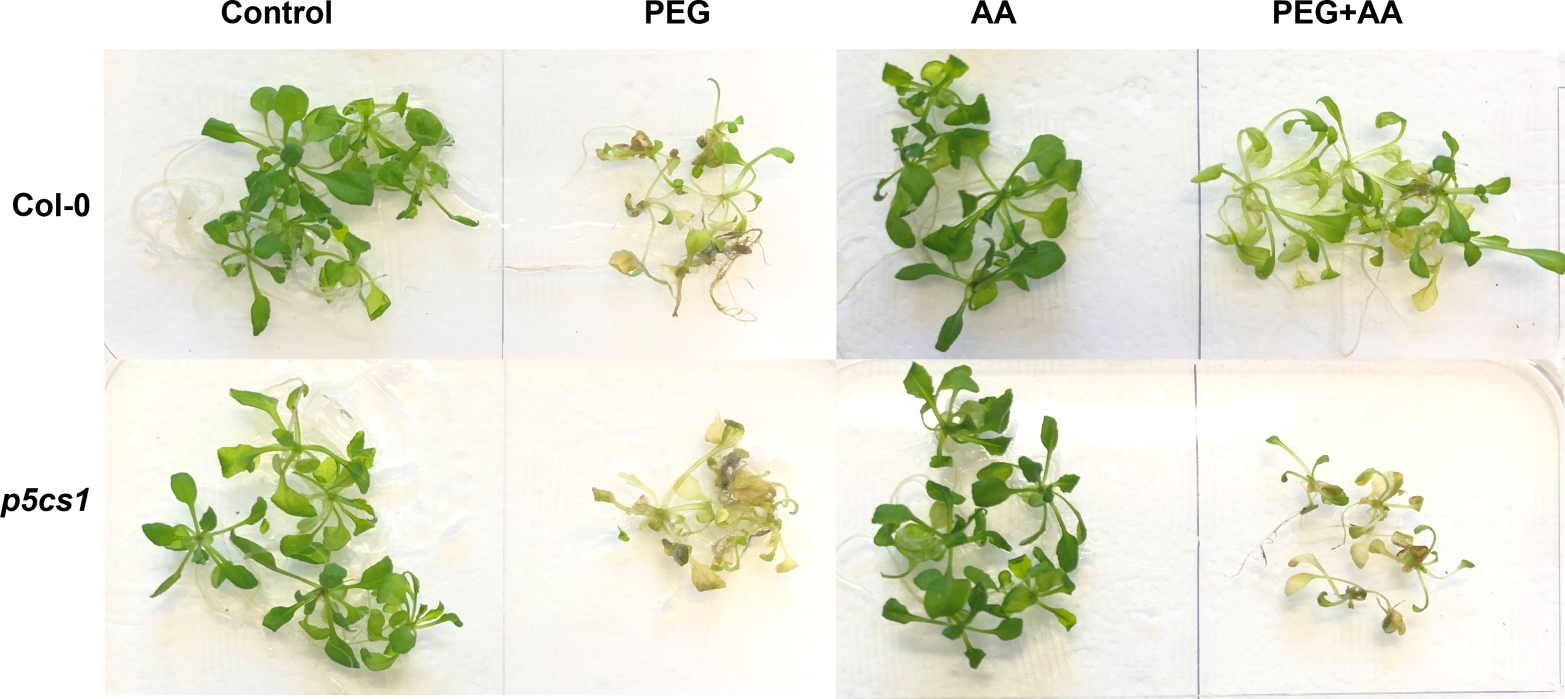
Phenotypic variation of Arabidopsis wild-type (Col-0) and *p5cs1* mutant line upon treatment with exogenous acetic acid under PEG treatments. 14 days old seedlings were subjected to exogenous treatments for 48 hours, brought back to the normal MS plate and subsequently grown for another seven days. Ctrl, PEG (20%), AA (500 μM), and PEG+AA represent treatments with dH_2_O, 20% polyethylene glycol, 500 μM acetic acid, and 20% polyethylene glycol + 500 μM acetic acid, respectively. FW, fresh weight. This photo is a representative of 3-time experiments.

As proline plays the potential role for acetic acid induced tolerance, we wanted to check the performance of the mutant disrupted in proline biosynthesis. We collected one T-DNA insertion line of proline biosynthetic gene, *P5CS1* and compared its performance with wild-type (Col-0) under the above-mentioned treatments. From the visual observations, no phenotypic differences were detected between ‘Ctrl’ and ‘AA’ (**Figure 7**). However, both wild-type and *p5cs1* mutant suffered terribly under ‘PEG’ and wilted to pale-colored leaves. Intriguingly, *p5cs1* mutant was negatively affected in ‘PEG+AA’. On the other hand, Col-0 plants still grew better than the others with slightly yellowish and narrower leaves (**Figure 7**). Overall, our data on Arabidopsis suggests a positive regulation of exogenous acetic acid on proline regulation to reduce the negative effects of drought stress.

## Discussion

Drought stress has negative consequences on leaf epinasty, stomatal closure, and photosynthesis, resulting in detrimental effects on plant physiology and growth. Plants have evolved adaptive mechanisms to survive under drought stress (Farooq et al., 2009; Gill and Tuteja, 2010; Claeys and Inze, 2013). Several reports suggested the positive effect of acetic acid in counteracting osmotic stress, especially caused by drought and salinity (Kim et al., 2017; Utsumi et al., 2019; Rahman et al., 2020). In our study, we employed two main approaches to find the potential roles of acetic acid in mitigation of PEG-induced osmotic effects, including i) priming of maize seeds with acetic acid (500 μM) and grown under PEG (20%) and ii) application of exogenous acetic acid (25 mM) to maize seedlings grown under PEG.

Seed priming with PEG resulted in stunted growth that was also consistent with the shoot and growth parameters. The stunted growth of maize plants was almost recovered in ‘PEG+AA’, although acetic acid alone did not exhibit any positive modulation (**Figure 1A**). We did not find any variation in the germination percentage of maize seeds among the treatments (**Supplementary Table 1**). Previous studies reported that germination rate of osmo-primed seeds was decreased in sorghum (Zhang et al., 2015) and in model plant *A. thaliana* (Alavilli et al., 2016). However, the stunted growth recovery in ‘PEG+AA’ might be due to stress memory transmission. This induced memory perhaps is transmitted from seed germination to seedling stage to induce the growth defects in ‘PEG’ and ultimately provided lesser canopy to combat the transpirational water loss. Several studies reported PEG- or water deficiency-induced shoot and root growth inhibition or growth parameters reduction while acetic acid application enhanced the recovery (Rahman et al., 2019; Utsumi et al., 2019; Rahman et al., 2020), which was also in parallel with our current findings (**Figures 1A, B**) Although the root length was much smaller, but the total root volume was higher in ‘PEG’ while both root length and root volume were almost similar in ‘PEG+AA’ compared with ‘Ctrl’ (**Table 1**). In corroboration with our findings, a previous study also suggested that the root tip growth was enhanced upon PEG treatment, resulting in a more enhanced diameter (Ji et al., 2014). However, similar root length and volume in ‘PEG+AA’ to ‘Ctrl’ suggest the alleviating capacity of acetic acid to overcome PEG-induced negative effects.

Variation was also observed for several leaf spectral indices, including NDVI, SPRI, MCARI, Ctr1, and Lic2 among different treatments. These indices are generally used to detect the effects of environmental stresses on different leaf parameters, such as leaf fluorescence and chlorophyll status (Li et al., 2018). In the current study, NDVI and SPRI had a decreasing trend while MCARI, Ctr1, and Lic2 increased under ‘PEG’ (**Table 1**). PEG-induced variation in the indices has not been explored comprehensively; however, our findings are well supported by recent findings on bread wheat under drought (Rustamova et al., 2021; Siddiqui et al., 2021). Increasing NDVI and SPRI, and decreasing MCARI, Ctr1, and Lic2 in ‘PEG+AA’ plants correlated with morphological parameters, indicating a growth promoting effect of acetic acid in maize (**Figures 1A, B**). NDVI and SPRI are broadband indices associated with plant biomass production and are often used to indirectly estimate the net primary productivity and photosynthetic capacity (Main et al., 2011; Begum et al., 2020). As stress index, intrinsic MCARI indicates the relative abundance of chlorophyll, generally a low MCARI value indicates high leaf chlorophyll content (Li et al., 2018), which was also observed in ‘PEG+AA’ compared with ‘PEG’ in the current study (**Table 1**). However, an increase in Ctr1 index suggests a change in reflectance, which might have occurred due to a shift in carotenoid to chlorophyll ratio (Penuelas et al., 1995). Under stress conditions, chlorophyll degrades at a faster rate compared to carotenoids (Penuelas et al., 1995; Liu et al., 2011), which partially substantiated stunted growth in ‘PEG’ even though no wilting symptoms were found (**Table 1**). Notably, the Lic2 index is significantly correlated with plant growth and grain yield and generally a lower value indicates high biomass (Begum et al., 2020) which was observed in ‘PEG+AA’ compared to ‘PEG’ (**Table 1**).

Alteration in leaf spectral indices in the root treated seedlings was not conspicuous albeit significant variation in three indices, including GI, PRI, and NPCI. GI and PRI were decreased in ‘PEG’ while remaining similar in ‘PEG+AA’ when compared with ‘Ctrl’ (**Supplementary Table 1**). On the other hand, NPCI followed the reciprocal trend (**Supplementary Table 1**). GI and PRI determine the activity of the photosynthetic apparatus under stress conditions (Gamon et al., 1997), which is in line with the wilting symptoms and low water content in ‘PEG’ plants while no changes recorded in ‘PEG+AA’ plants (**Figures 2A, B**). An increase in NPCI often indicates chlorophyll degradation (Peñuelas et al., 1994), and thus the increase of NPCI in ‘PEG’ plants and no change in ‘PEG+AA’ plants highlighted the control of acetic acid over photosynthetic capacity in maize. Moreover, results of gas exchange parameters revealed that PEG-mediated stress perturbed photosynthetic performance with reduced photosynthetic rate and net carbon content, which also coincided with the findings related to severe wilting symptoms (**Figure 1C**). In contrast, an improved photosynthetic rate in ‘PEG+AA’ plants despite having declined stomatal conductance and reduced transpiration rate (**Figure 3**) indicated an intrinsic mechanism employed by acetic acid to sustain maize photosynthetic ability under water-shortage conditions. Similar findings were also reported mung bean plants under salinity stress (Rahman et al., 2019). It is likely that higher transpiration rate and subsequent loss of leaf turgidity were responsible for greater susceptibility to PEG-mediated osmotic stress. By contrast, reduced transpiration rate and stomatal conductance were observed in ‘PEG+AA’ plants, which coincided with higher RWC and less wilting (**Figures 2A, B**). Overall, it is pertinent that acetic acid application adjusted the balance between transpirational loss and photosynthetic capacity, which ultimately led to enhanced tolerance to PEG-mediated osmotic stress.

A vibrant antioxidant system is pre-requisite to detoxify excessive ROS under different types of abiotic stress (Sade et al., 2011; Choudhury et al., 2017; Sehar et al., 2021). In this study, significant increase in MDA and H_2_O_2_ were observed in ‘PEG’ plants in comparison with ‘Ctrl’ plants, suggesting that PEG treatment led to a generation of oxidative stress (**Figures 4A, B**). On the other hand, reduction of MDA and H_2_O_2_ contents was positively correlated with the enhanced activities of antioxidant enzymes, including CAT, POD and APX in ‘PEG+AA’ plants (**Figure 4C**). These results imply that acetic acid boosted maize antioxidant mechanism by enhancing the activities of key antioxidant enzymes, which ultimately contributed to the alleviation of drought-caused oxidative stress. Intriguingly, proline content was elevated even under acetic acid treatment alone (**Figure 5A**). Although there was no variation in the H_2_O_2_ and MDA contents in priming treatments, the amount of proline was increased in ‘PEG+AA’ plants (**Figure 1D**), suggesting that stimulation of proline accumulation may be a putative function of acetic acid to fight against drought stress in plants. Like our findings, mung bean plants treated with acetic acid in presence or absence of salt stress displayed an enhanced accumulation of endogenous proline in the leaves (Rahman et al., 2019). It is likely that the lower level of proline in the ‘PEG’ plants was not sufficient to surpass the PEG-induced osmotic effects, eventually aggravating oxidative damage in maize. Our RT-qPCR data also showed that the enhanced transcript levels of *ZmP5CS1* positively correlated with proline accumulation in ‘PEG+AA’ plants (**Figures 1D**, **5B**). These results suggest that acetic acid regulates proline level by upregulating the expression of *P5CS1* in plants. We further examined the role of acetic acid in proline metabolism by investigating the drought tolerance of wild-type and *p5cs1* plants of Arabidopsis. PEG-treatment resulted in severe damage in both wild-type and *p5cs1* plants, while under acetic acid application only wild-type survived through severe drought stress but *p5cs1* exhibited profound damage and less recovery (**Figure 7**). These results underpin that the inability of *p5cs1* to perceive acetic acid treatment through proline accumulation resulted in severe damage under drought stress. Indeed, these results were well-supported by our findings on maize plants primed and treated with acetic acid during seed germination and seedling developments, respectively. Based on this, it appears that acetic acid-mediated high proline accumulation aided maize plants overcoming drought effects.

## Conclusions

The present study provides a mechanistic insight into the role of acetic acid in mediating PEG-induced drought tolerance in maize. Drought stress mitigation strategy of acetic acid application was plausibly correlated with the accumulation of endogenous proline through the upregulation of *P5CS1* in plants. Our complementary study using proline biosynthetic mutant *p5cs1* provided additional evidence on the regulatory roles of acetic acid on proline biosynthesis and accumulation under osmotic stress conditions in plants. The overexpression of *P5CS1* upon AA treatment could also underly other metabolic mechanisms associated to the formation of the product P5C that is highly linked to the glutamate and ornithine pathways. Although rigorous study is still needed for proper understanding of acetic acid-mediated drought tolerance, the present study underpinned a vital clue on the potential molecular mechanism of acetic acid in alleviating osmotic stress effects on plants. Because acetic acid is cheap, ecofriendly, and easily accessible, it could provide a dramatic solution for management of drought stress effects on plants in drought-prone areas worldwide.

## Data availability statement

The original contributions presented in the study are publicly available. This data can be found here: https://www.ncbi.nlm.nih.gov/nuccoreMaize: 19 α-zein (NM_001152088), P5CS1(NM_001352327). Arabidopsis: Actin2 (At3g18780), AtP5CS1 (At2g39800).

## Author contributions

SM designed the research. SM and MK performed the research. SM and MM analyzed the data and prepared the figures. KA, DAM, and MK acquired funding. SM, MM, and MK wrote the paper with the input from all authors. All authors contributed to the article and approved the submitted version.

## Supplementary material

Supplementary Figure 2 Dose-specific effect of acetic acid on Arabidopsis seed germination and proline content. **(A, B)** Germination percentage calculated **(A)** and the level of proline content **(B)** from the 3 days old germinated seedlings after plating them into MS medium containing **(A)** or treating 48 h with **(B)** different concentration of acetic acid. Data represent mean ± SE of three individual replicates (n=3) with 100 individual seeds for germination percentage and six individual replicates (n=6) for proline content, and different alphabetical letters indicate significant variations among the treatments following Tukey’s *Post-Hoc* HSD test (*P*<0.05).

## References

[B1] Adamipour N. Khosh-Khui M. Salehi H. Razi H. Karami A. Moghadam A. (2020). Metabolic and genes expression analyses involved in proline metabolism of two rose species under drought stress. Plant Physiol. Biochem. 155, 105–113. doi: 10.1016/j.plaphy.2020.07.028 32745929

[B2] Alavilli H. Awasthi J. P. Rout G. R. Sahoo L. Lee B. H. Panda S. K. (2016). Overexpression of a barley aquaporin gene, HvPIP2; 5 confers salt and osmotic stress tolerance in yeast and plants. Front. Plant Sci. 7. doi: 10.3389/fpls.2016.01566 PMC5073208 27818670

[B3] Alvarez M. E. Savouré A. Szabados L. (2022). Proline metabolism as regulatory hub. Trends Plant Sci. 27 (1), 39–55. doi: 10.1016/j.tplants.2021.07.009 34366236

[B4] Avramova V. Elgawad H. Zhang Z. Fotschki B. Casadevall R. Vergauwen L. (2015). Drought induces distinct growth response, protection, and recovery mechanisms in the maize leaf growth zone. Plant Physiol. 169, 1382–1396. doi: 10.1104/pp.15.00276 26297138 PMC4587441

[B5] Badr A. El-Shazly H. H. Tarawneh R. A. Börner A. (2020). Screening for drought tolerance in maize (*Zea mays* l.) germplasm using germination and seedling traits under simulated drought conditions. Plants 9, 565. doi: 10.3390/plants9050565 32365550 PMC7284379

[B6] Bates L. S. Waldren R. P. Teare I. D. (1973). Rapid determination of free proline for water-stress studies. Plant Soil. 39, 205–207. doi: 10.1007/BF00018060

[B7] Begum H. Alam M. S. Feng Y. Koua P. Ashrafuzzaman M. Shrestha A.. (2020). Genetic dissection of bread wheat diversity and identification of adaptive loci in response to elevated tropospheric ozone. Plant Cell Environ. 43, 2650–2665. doi: 10.1111/pce.13864 32744331

[B8] Chen C. Cui X. Zhang P. Wang Z. Zhang J. (2021). Expression of the pyrroline-5-carboxylate reductase (P5CR) gene from the wild grapevine *Vitis yeshanensis* promotes drought resistance in transgenic *Arabidopsis.* plant physiol. Biochem. 168, 188–201. doi: 10.1016/j.plaphy.2021.10.004 34649022

[B9] Choudhury F. K. Rivero R. M. Blumwald E. Mittler R. (2017). Reactive oxygen species, abiotic stress and stress combination. Plant J. 90, 856–867. doi: 10.1111/tpj.13299 27801967

[B10] Chun S. C. Paramasivan M. Chandrasekaran M. (2018). Proline accumulation influenced by osmotic stress in arbuscular mycorrhizal symbiotic plants. Front. Plant Sci. 9. doi: 10.3389/fmicb.2018.02525 PMC6232873 30459731

[B11] Claeys H. Inze D. (2013). The agony of choice: how plants balance growth and survival under water limiting conditions. Plant Physiol. 162, 1768–1779. doi: 10.1104/pp.113.220921 23766368 PMC3729759

[B12] Daughtry C. S. T. Walthall C. L. Kim M. S. Brown E. C. McMurtrey J. E. (2000). Estimating corn leaf chlorophyll concentration from leaf and canopy reflectance. Remot. Sens. Env. 74, 229–239. doi: 10.1016/S0034-4257(00)00113-9

[B13] de Mendiburu F. (2019) Agricolae: statistical procedures for agricultural research. r package version 1. Available at: https://CRAN.R-project.org/package=agricolae.

[B14] Fang L. Yin X. Peter E. L. Martre P. Struik P. C. (2022). Drought exerts a greater influence than growth temperature on the temperature response of leaf day respiration in wheat (*Triticum aestivum*). Plant Cell Environ. 45, 2062–2077. doi: 10.1111/pce.14324 35357701 PMC9324871

[B15] Farooq M. Hussain M. Wakeel A. Siddiqui K. H. M. (2015). Salt stress in maize: effects, resistance mechanisms, and management: a review. Agron. Sustain. Dev. 35, 461–481. doi: 10.1007/s13593-015-0287-0

[B16] Farooq M. Wahid A. Kobayashi N. Fujita D. Barsa S. M. A. (2009). Plant drought stress: effects, mechanisms and management. Agron. Sustain. Dev. 29, 185–212. doi: 10.1051/agro:2008021

[B17] Funck D. Winter G. Baumgarten L. Forlani G. (2012). Requirement of proline synthesis during *Arabidopsis* reproductive development. BMC Plant Biol. 12, 191. doi: 10.1186/1471-2229-12-191 23062072 PMC3493334

[B18] Gamon J. Serrano L. Surfus J. (1997). The photochemical reflectance index: an optical indicator of photosynthetic radiation use efficiency across species, functional types, and nutrient levels. Oecologia. 112, 492–501. doi: 10.1007/s004420050337 28307626

[B19] Ghoulam C. Fours A. Fares K. (2002). Effects of salt stress on growth, inorganic ions and proline accumulation in relation to osmotic adjustment in five sugar beet cultivars. Environ. Exp. Bot. 47, 39–50. doi: 10.1016/S0098-8472(01)00109-5

[B20] Gill S. S. Tuteja N. (2010). Reactive oxygen species and antioxidant machinery in abiotic stress tolerance in crop plants. J. Plant Physiol. 48, 909–930. doi: 10.1016/j.plaphy.2010.08.016 20870416

[B21] Gupta A. Rico-Medina A. Cano-Delgado A. I. (2020). The physiology of plant responses to drought. Science 368, 266–269. doi: 10.1126/science.aaz7614 32299946

[B22] Hemeda H. M. Klein B. P. (1990). Effects of naturally occurring antioxidants on peroxidase activity of vegetable extracts. J. Food Sci. 55, 184 185. doi: 10.1111/j.1365-2621.1990.tb06048.x

[B23] Hodges D. DeLong J. Forney C. Prange R. K. (1999). Improving the thiobarbituric acid-reactive-substances assay for estimating lipid peroxidation in plant tissues containing anthocyanin and other interfering compounds. Planta. 207, 604–611. doi: 10.1007/s00425-017-2699-3 28456836

[B24] Ibrahim A. A. Mageed A. E. T. Abohamid Y. Abdallah H. El-Saadony M. AbuQamar S. (2022). Exogenously applied proline enhances morph-physiological responses and yield of drought-stressed maize plants grown under different irrigation systems. Front. Plant Sci. 13. doi: 10.3389/fpls.2022.897027 PMC9331896 35909786

[B25] Ji H. Liu L. Li K. Xie Q. Wang Z. Zhao X.. (2014). PEG-mediated osmotic stress induces premature differentiation of the root apical meristem and outgrowth of lateral roots in wheat. J. Exp. Bot. 65, 4863–4872. doi: 10.1093/jxb/eru255 24935621 PMC4144773

[B26] Kamruzzaman M. Beyene M. A. Siddiqui M. N. Ballvora A. Léon J. Naz A. A. (2022). Pinpointing genomic loci for drought-induced proline and hydrogen peroxide accumulation in bread wheat under field conditions. BMC Plant Biol. 22, 584. doi: 10.1186/s12870-022-03943-9 36513990 PMC9746221

[B27] Kang J. Peng Y. Xu W. (2022). Crop root responses to drought stress: molecular mechanisms, nutrient regulations, and interactions with microorganisms in the rhizosphere. Int. J. Mol. Sci. 23, 9310. doi: 10.3390/ijms23169310 36012575 PMC9409098

[B28] Kim J. M. To T. Matsui A. Tanoi K. Kobayashi N. I. Matsuda F.. (2017). Acetate-mediated novel survival strategy against drought in plants. Nat. Plants. 3, 17097. doi: 10.1038/nplants.2017.97 28650429

[B29] Lei C. Bagavathiannan M. Wang H. Sharpe S. M. Meng W. Yu J. (2021). Osmopriming with polyethylene glycol (PEG) for abiotic stress tolerance in germinating crop seeds: a review. Agron. 11, 2194. doi: 10.3390/agronomy11112194

[B30] Li M. Chu R. Yu Q. Islam A. R. M. Chou S. Shen S. (2018). Evaluating structural, chlorophyll-based and photochemical indices to detect summer maize responses to continuous water stress. Water 10, 500. doi: 10.3390/w10040500

[B31] Liu C. C. Liu Y. G. Guo K. Fan D. Y. Li G. G. Zheng Y.. (2011). Effect of drought on pigments, osmotic adjustment and antioxidant enzymes in six woody plant species in karst habitats of south- western China. Env. Exp. Bot. 71, 174–183. doi: 10.1016/j.envexpbot.2010.11.012

[B32] Mahmud S. Ullah C. Kortz A. Bhattacharyya S. Yu P. Gershenzon J.. (2022). Constitutive expression of *JASMONATE RESISTANT 1* induces molecular changes that prime the plants to better withstand drought. Plant Cell Environ. 45, 2906–2922. doi: 10.1111/pce.14402 35864601

[B33] Main R. Cho M. A. Mathieu R. O’Kennedy M. M. Ramoelo A. Koch S. (2011). An investigation into robust spectral indices for leaf chlorophyll estimation. ISPRS J. Phot. Remo. Sens. 66, 751–761. doi: 10.1016/j.isprsjprs.2011.08.001

[B34] Mittler R. Zandalinas S. I. Fichman Y. Breusegem F.V. (2022). Reactive oxygen species signalling in plant stress responses. Nat. Rev. Mol. Cell. Biol. 23, 663–679. doi: 10.1038/s41580-022-00499-2 35760900

[B35] Mosaad I. S. M. Ayman H. I. S. Moustafa-Farag M. Seadh A. K. (2020). Effect of exogenous proline application on maize yield and the optimum rate of mineral nitrogen under salinity stress, J. Plant Nutr. 43, 354–370. doi: 10.1080/01904167.2019.1676901

[B36] Moukhtari A. Cabassa-Hourton C. Farissi M. Savoure A. (2020). How does proline treatment promote salt stress tolerance during crop plant development? Front. Plant Sci. 11. doi: 10.3389/fpls.2020.01127 PMC7390974 32793273

[B37] Nakano Y. Asada K. (1981). Hydrogen peroxide is scavenged by ascorbate-specific peroxidase in spinach chloroplasts. Plant Cell Physiol. 22, 867–880. doi: 10.1093/oxfordjournals.pcp.a076232

[B38] Nikoleta-Kleio D. Theodoros D. Roussos P. A. (2020). Antioxidant defense system in young olive plants against drought stress and mitigation of adverse effects through external application of alleviating products. Sci. Hortic. 259, 108812. doi: 10.1016/j.scienta.2019.108812

[B39] Ogawa D. Suzuki Y. Yokoo T. Katoh E. Teruya M. Muramatsu M.. (2021). AA-acid-induced jasmonate signaling in root enhances drought avoidance in rice. Sci. Rep. 11, 6280. doi: 10.1038/s41598-021-85355-7 33737547 PMC7973560

[B40] Ozturk M. Turkyilmaz U. B. García-Caparrós P. Khursheed A. Gul A. Hasanuzzaman M. (2021). Osmoregulation and its actions during the drought stress in plants. Physol. Plant 172, 1321–1335. doi: 10.1111/ppl.13297 33280137

[B41] Peñuelas J. Gamon J. Fredeen A. Merino J. Field C. (1994). Reflectance indices associated with physiological changes in nitrogen-and water-limited sunflower leaves. Remote Sens. Environ. 48, 135–146. doi: 10.1016/0034-4257(94)90136-8

[B42] Penuelas J. Baret F. Filella I. (1995). Semiempirical indexes to assess carotenoids chlorophyll-a ratio from leaf spectral reflectance. Photosynthetica 31, 221–230.

[B43] Rahman M. Mostofa M. G. Keya S. S. Rahman A. Das A. K Islam R.. (2020). Acetic acid improves drought acclimation in soybean: an integrative response of photosynthesis, osmoregulation, mineral uptake and antioxidant defense. Physiol. Plant 172, 334–350. doi: 10.1111/ppl.13191 32797626

[B44] Rahman M. M. Mostofa M. G. Rahman M. A. Islam M.R. Keya S.S. Das A.K.. (2019). Acetic acid: a cost-effective agent for mitigation of seawater-induced salt toxicity in mung bean. Sci. Rep. 9, 15186. doi: 10.1038/s41598-019-51178-w 31645575 PMC6811677

[B45] Rivero L. Scholl R. Holomuzki N. Crist D. Grotewold E. Brkljacic J. (2013). Handling *Arabidopsis* plants: growth, preservation of seeds, transformation, and genetic crosses. Methods Mol. Biol. 1062, 3–25. doi: 10.1007/978-1-62703-580-4_1 24057358

[B46] Rouse J. W. Haas R. H. Schell J. A. Deering D. W. (1973). “Monitoring vegetation systems in the great plains with ERTS,” in Proceedings of 3rd earth resources technology satellite symposium, Greenbelt, SP-351. 309–317.

[B47] Rustamova S. Shreshtha A. Naz A. A. Huseynova I. (2021). Expression profiling of DREB1 and evaluation of vegetation indices in contrasting wheat genotypes exposed to drought stress. Plant Gene. 25, 266. doi: 10.1016/j.plgene.2020.100266

[B48] Sade B. Soylu S. Yetim E. (2011). Drought and oxidative stress. Afric. J. Biotech. 10, 11102–11109. doi: 10.5897/AJB11.1564

[B49] Salika R. Rifat J. (2021). Abiotic stress responses in maize: a review. Acta Physiol. Plant 43, 130. doi: 10.1007/s11738-021-03296-0

[B50] Sehar Z. Jahan B. Masood A. Anjum N. A. Khan N. A. (2021). Hydrogen peroxide potentiates defense system in presence of sulfur to protect chloroplast damage and photosynthesis of wheat under drought stress. Physiol. Planta. 172, 922–934. doi: 10.1111/ppl.13225 32997365

[B51] Sergiev I. Alxieva V. Karanov E. (1997). Effect of spermone, atrazine and combination between them on some endogenous protective systems and stress markers in plants. Comp. Rend. Acad. Bulg. Sci. 51, 121–124.

[B52] Sheoran S. Kaur Y. Kumar S. Shukla S. Rakshit S. Kumar R. (2022). Recent advances for drought stress tolerance in maize (*Zea mays* l.): present status and future prospects. Front. Plant Sci. 13. doi: 10.3389/fpls.2022.872566 PMC9189405 35707615

[B53] Shrestha A. Cudjoe D. K. Kamruzzaman M. Siddique S. Fiorani F. Léon J.. (2021). Abscisic acid-responsive element binding transcription factors contribute to proline synthesis and stress adaptation in *Arabidopsis.* J. Plant Physiol. 261, 153414. doi: 10.1016/j.jplph.2021 33895677

[B54] Siddiqui M. N. Teferi T. J. Ambaw A. M. Gabi M. T. Koua P. Léon J.. (2021). New drought-adaptive loci underlying candidate genes on wheat chromosome 4B with improved photosynthesis and yield responses. Physiol. Planta. 173, 2166–2180. doi: 10.1111/ppl.13566 34549429

[B55] Singh C. K. Rajkumar B. K. Kumar V. (2021). Differential responses of antioxidants and osmolytes in upland cotton (*Gossypium hirsutum*) cultivars contrasting in drought tolerance. Plant Stress. 2, 100031. doi: 10.1016/j.stress.2021.100031

[B56] Spormann S. Nadais P. Sousa F. Pinto M. Martins M. Sousa B.. (2023). Accumulation of proline in plants under contaminated soils–are we on the same page? Antioxidants 12 (3), 666. doi: 10.3390/antiox12030666 36978914 PMC10045403

[B57] Utsumi Y. Chikako U. Maho T. Chien V. H. Takahashi S. Matsui A.. (2019). Acetic acid treatment enhances drought avoidance in cassava (*Manihot esculenta*). Front. Plant Sci. 10, 521. doi: 10.3389/fpls.2019.00521 31105723 PMC6492040

[B58] Velikova V. Yordanov I. Edreva A. (2000). Oxidative stress and some antioxidant systems in acid rain-treated bean plants: protective role of exogenous polyamines. Plant Sci. 151, 59–66. doi: 10.1016/S0168-9452(99)00197-1

[B59] Wickham H. Averick M. Bryan J. Chang W. McGowan L.D. François R. (2021). Wecome to tydyverse. J. Open Source Softw. 4, 1686. doi: 10.21105/joss.01686

[B60] Zhang F. Yu J. Johnston C. R. Wang Y. Zhu K. Lu F.. (2015). Seed priming with polyethylene glycol induces physiological changes in sorghum (*Sorghum bicolor* l. moench) seedlings under suboptimal soil moisture environments. PloS One 10, 10. doi: 10.1371/journal.pone.0140620 PMC4607411 26469084

