# Supplementary material for: Acetic acid positively modulates proline metabolism for mitigating PEG-mediated drought stress in Maize and Arabidopsis
Source: Front Plant Sci. 2023 Jul 19;14:1167238. doi: 10.3389/fpls.2023.1167238 (PMC10394635; doi:10.3389/fpls.2023.1167238)
Supplement: Supplementary Figure 2 — Dose-specific effect of acetic acid on Arabidopsis seed germination and proline content. (A, B) Germination percentage calculated (A) and the level of proline content (B) from the 3 days old germinated seedlings after plating them into MS medium containing (A) or treating 48 h with (B) different concentration of acetic acid. Data represent mean ± SE of three individual replicates (n=3) with 100 individual seeds for germination percentage and six individual replicates (n=6) for proline content, and different alphabetical letters indicate significant variations among the treatments following Tukey’s Post-Hoc HSD test (P<0.05). [file DataSheet_1.zip › Data Sheet 1 (3).docx]

**Supplementary Information**

**Supplementary Table 1:** Effect of acetic acid on leaf spectral reflectance indices of maize plants subjected to PEG treatments*****

| **Parameter** | | **Ctrl** | **PEG** | **AA** | **PEG+AA** |
| --- | --- | --- | --- | --- | --- |
| **GI** | Mean | 1.745^a^ | 1.447^b^ | 1.709^a^ | 1.717^a^ |
|  | SD | 0.142 | 0.133 | 0.037 | 0.049 |
| **PRI** | Mean | 0.0459^a^ | 0.0136^c^ | 0.0429^ab^ | 0.0397^b^ |
|  | SD | 0.013 | 0.0013 | 0.003 | 0.002 |
| **NPCI** | Mean | 0.0351^b^ | 0.065^a^ | 0.034^b^ | 0.032^b^ |
|  | SD | 0.0154 | 0.0159 | 0.003 | 0.001 |

* Data represented as mean, standard deviation (SD) of six individual replicates along with the alphabetical letters representing different levels of statistical significance calculated with one-way ANOVA and Tukey‘s Post-Hoc HSD test (*P*< 0.05). GI, Greenness Index; PRI, Photochemical Reflectance Index; NPCI, Normalized Pigment Chlorophyll Index.

**Supplementary Table 2: List of primers used for RT-qPCR in this study.**

| **Gene name** | **Primer Sequence** |
| --- | --- |
| *Arabidopsis P5CS1*_fw | AAGAGCCCCATATCAGGATTCTTCT |
| *Arabidopsis P5CS1*_rev | TGTGTAAAGACCTTCAACATCGCTC |
| *Arabidopsis Actin2*_fw | TGCCAATCTACGAGGGTTTC |
| *Arabidopsis Actin2*_rev | CTTACAATTTCCCGCTCTGC |
| Maize *P5CS1*_fw | GCACTCTTGCACGTATGGAA |
| Maize *P5CS1*_rev | GCCACTTCGAATCGCTAAAG |
| Maize *19 α-zein*_fw | GCTCCTTGGTCTTTCTGCAA |
| Maize *19 α-zein*_rev | GGTAACTGCTGTAATAGGGCTGATG |


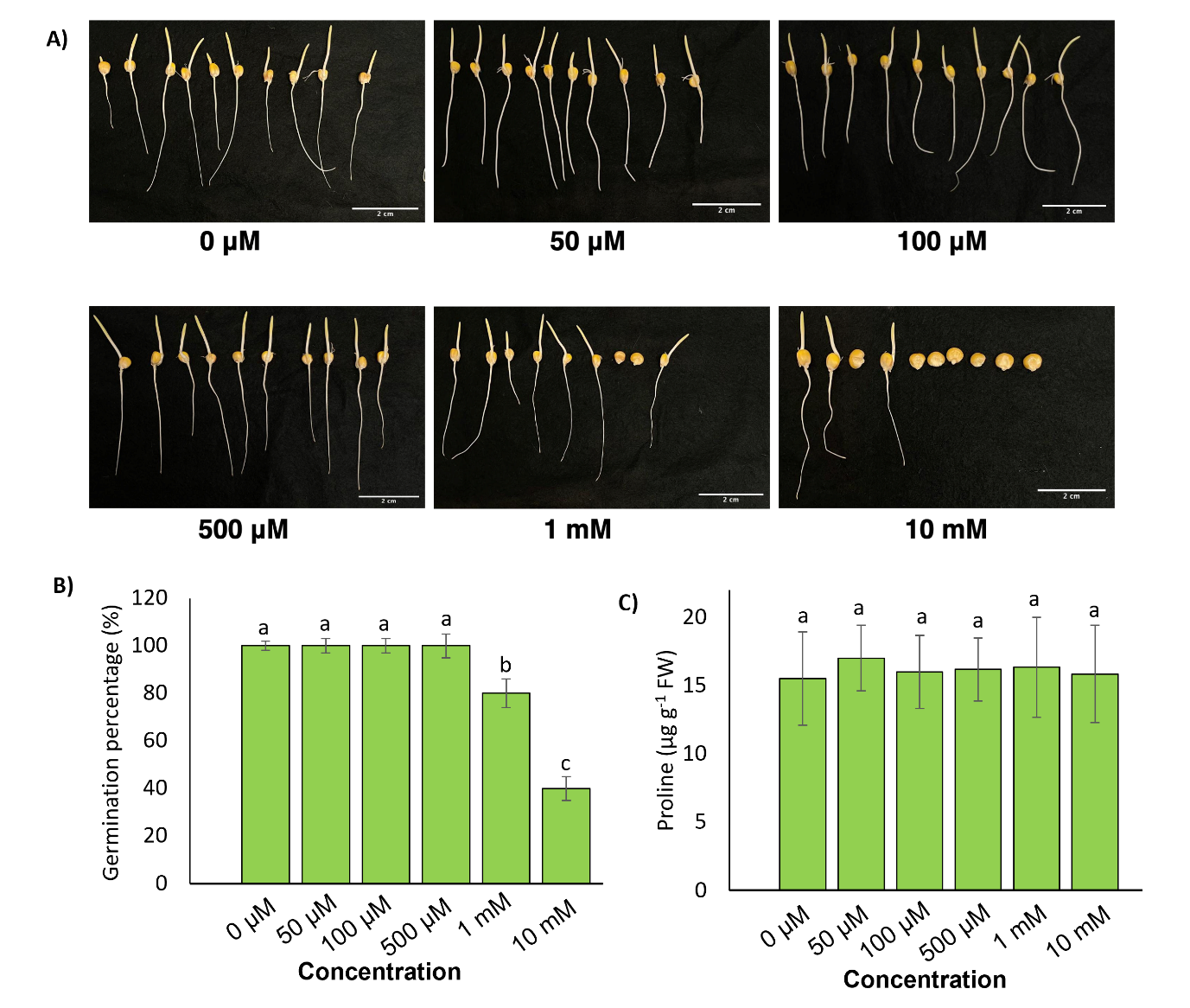


**Supplementary Figure 1:** Dose-specific priming effect of acetic acid on maize seed

germination and proline content. (A-B) Representative photos taken (A) and germination

percentage calculated (B) from the germinated seedling after priming with different

concentration of acetic acid. Seeds were primed with the indicated concentrations of acetic

acid overnight and placed them in a dark growth chamber for 48 h. (C) The level of proline

content in 10 days old maize leaves recorded after seed priming with different concentration

of acetic acid. Data represent mean ± SE of three individual replicates (n=3) with 20

individual seeds for germination percentage and six individual replicates (n=6) for proline

content, and different alphabetical letters indicate significant variations among the treatments

following Tukey‘s Post-Hoc HSD test (*P*<0.05).


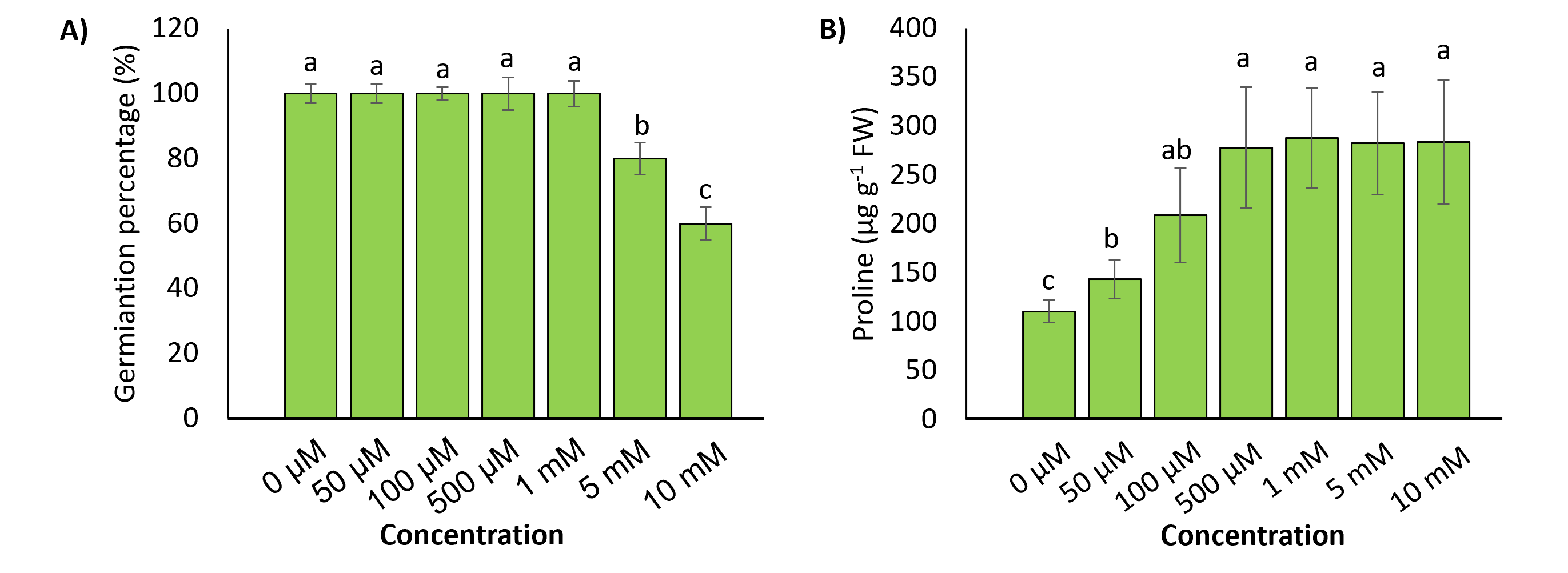


**Supplementary Figure 2:** Dose-specific effect of acetic acid on Arabidopsis seed germination and proline content. (A, B) Germination percentage calculated (A) and the level of proline content (B) from the 3 days old germinated seedlings after plating them into MS medium containing (A) or treating 48 h with (B) different concentration of acetic acid. Data represent mean ± SE of three individual replicates (n=3) with 100 individual seeds for germination percentage and six individual replicates (n=6) for proline content, and different alphabetical letters indicate significant variations among the treatments following Tukey’s Post-Hoc HSD test (P<0.05).
